# The Effect of Liquid-Phase Exfoliated Graphene Film on Neurodifferentiation of Stem Cells from Apical Papilla

**DOI:** 10.3390/nano12183116

**Published:** 2022-09-08

**Authors:** Jelena Simonovic, Bosko Toljic, Milos Lazarevic, Maja Milosevic Markovic, Mina Peric, Jasna Vujin, Radmila Panajotovic, Jelena Milasin

**Affiliations:** 1 School of Dental Medicine, University of Belgrade, 11000 Belgrade, Serbia; 2 Center for Laser Microscopy, Faculty of Biology, University of Belgrade, 11000 Belgrade, Serbia; 3 Graphene Laboratory, Center for Solid State Physics and New Materials, Institute of Physics, University of Belgrade, 11000 Belgrade, Serbia

**Keywords:** graphene, dental stem cells, stem cells from apical papilla, neurogenic differentiation

## Abstract

Background: Dental stem cells, which originate from the neural crest, due to their easy accessibility might be good candidates in neuro-regenerative procedures, along with graphene-based nanomaterials shown to promote neurogenesis *in vitro*. We aimed to explore the potential of liquid-phase exfoliated graphene (LPEG) film to stimulate the neuro-differentiation of stem cells from apical papilla (SCAP). Methods: The experimental procedure was structured as follows: (1) fabrication of graphene film; (2) isolation, cultivation and SCAP stemness characterization by flowcytometry, multilineage differentiation (osteo, chondro and adipo) and quantitative PCR (qPCR); (3) SCAP neuro-induction by cultivation on polyethylene terephthalate (PET) coated with graphene film; (4) evaluation of neural differentiation by means of several microscopy techniques (light, confocal, atomic force and scanning electron microscopy), followed by neural marker gene expression analysis using qPCR. Results: SCAP demonstrated exceptional stemness, as judged by mesenchymal markers’ expression (CD73, CD90 and CD105), and by multilineage differentiation capacity (osteo, chondro and adipo-differentiation). Neuro-induction of SCAP grown on PET coated with graphene film resulted in neuron-like cellular phenotype observed under different microscopes. This was corroborated by the high gene expression of all examined key neuronal markers (Ngn2, NF-M, Nestin, MAP2, MASH1). Conclusions: The ability of SCAPs to differentiate toward neural lineages was markedly enhanced by graphene film.

## 1. Introduction

Regenerative medicine aims at replacing damaged human cells, tissues or organs and restoring their normal architecture and functions [1]. Stem cells (SCs) emerged as a promising tool in regenerative therapies due to their ability to differentiate into numerous cell lineages, high self-renewal capacity and immunosuppressive activity. A variety of new materials and new devices, enhancing cell migration, proliferation, and differentiation, have been developed as well [2,3].

Since SC research has dramatically evolved over the past years, it is possible now to isolate SCs from almost any tissue [4,5,6,7]. Yet, in many instances, the most appropriate and matching source of stem cells for a given regenerative therapy remains to be identified.

Dental SCs share a similar origin as neuronal stem cells, as they originate from the neural crest, and due to their accessibility and absence of ethical issues, they might be a good candidate for neuro-regeneration. Apical papilla is a soft tissue at the apex of a not fully formed tooth, containing more than 95% of mesenchymal SCs (stem cells from apical papilla, SCAP) [8,9]. SCAP express some early neural markers even without neural induction and can be transformed into different cell types belonging to neural lineage [10], making them suitable for potential therapeutic applications in different clinical settings necessitating neuro-repair. SCAP differentiation potential has been extensively tested, but mainly in experiments of osteogenesis and odontogenesis. Only a few studies have dealt with the use of SCAP in neurodifferentiation. For instance, it was shown that fibrin gels [11] and hypoxia [12] stimulate SCAP neurogenesis.

Graphene, an allotrope of carbon, owing to its physico-chemical and biological properties, is also becoming increasingly popular in bioengineering [13,14,15,16,17]. Graphene and graphene-based nanomaterials (GBN), especially graphene oxide, improve cell adhesion during proliferation and differentiation and, due to their electrical conductivity, have the ability to promote the process of differentiation towards neural cells [18,19,20,21,22,23,24,25,26]. Furthermore, a colloidal dispersion of graphene demonstrated excellent biocompatibility, nontoxicity and remarkable support for cell proliferation [27,28,29,30,31].

As already stated, in numerous studies focusing on tissue engineering, graphene-based materials have been used in conjunction with different dental stem cells, such as dental pulp stem cells, periodontal ligament stem cells and dental follicle stem cells (reviewed by Guazzo et al. [32]). However, differentiation experiments involving graphene derivatives and stem cells from apical papilla are extremely scarce.

Given the lack of studies on SCAP biological behavior when in contact with graphene film, we sought to explore, by means of different microscopy techniquesand real-time gene expression analyses, the potential of liquid-phase exfoliated graphene (LPEG) film to induce and stimulate the neuro-differentiation of SCAP.

## 2. Materials and Methods

**Figure 1:**
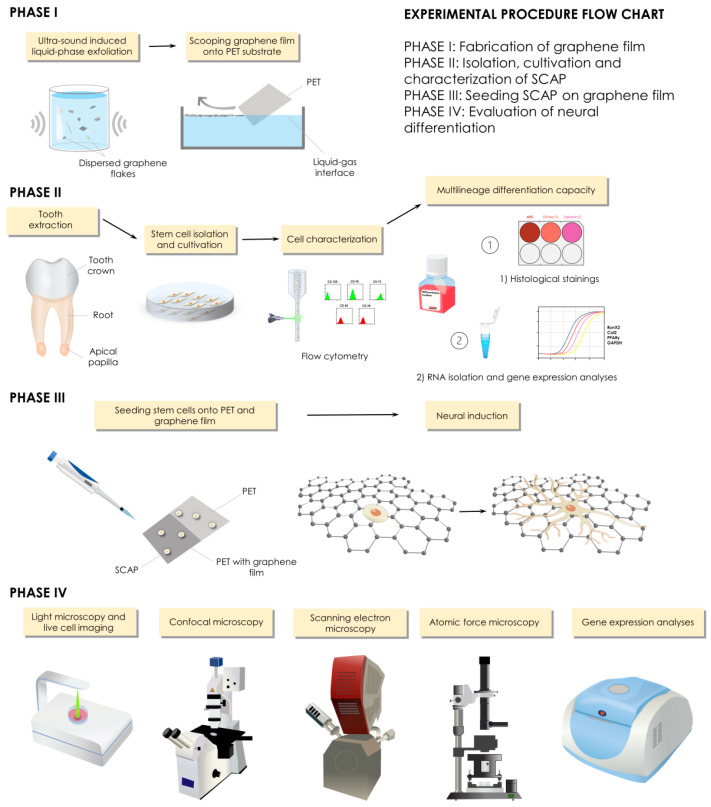
Study design and experimental procedures.

The experimental procedure was structured into four phases: phase 1—fabrication of graphene film; phase 2—isolation, cultivation and characterization of stem cells derived from apical papilla; phase 3—seeding stem cells on graphene film and PET; phase 4—evaluation of neural differentiation (Figure 1).

### 2.1. Fabrication of Graphene Film

#### 2.1.1. Preparation of Graphene Dispersion

**Figure 2:**
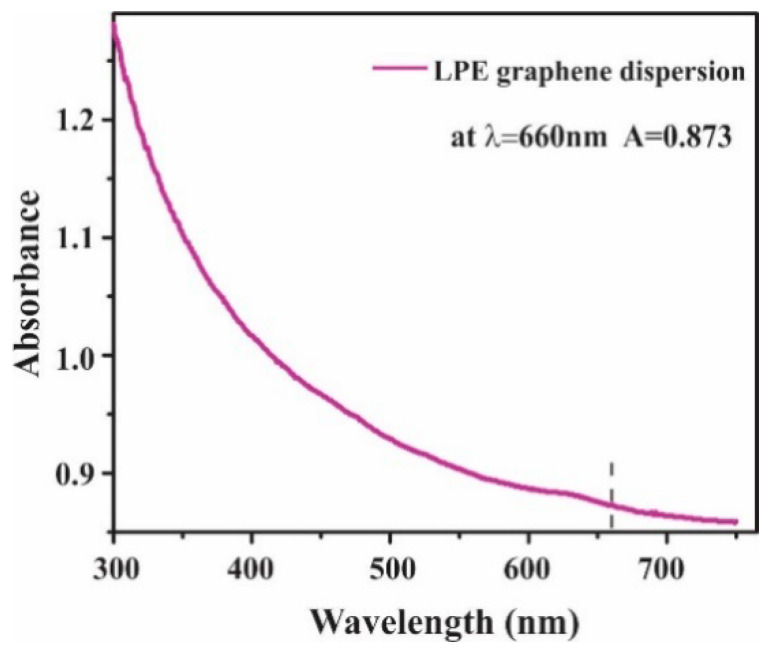
UV-VIS absorption spectrum of LPE graphene dispersion.

The graphene dispersion utilized in this study was prepared by the liquid-phase exfoliation method (LPE) [33]. Following the procedure described in our previous work [34], the mixture was made by adding the graphite powder (Sigma Aldrich-332461) in N-Methyl-2-pyrrolidone (NMP, Sigma Aldrich-328634). The initial concentration was 18 mg/mL. The solution was exposed to ultrasound (Sonic bath, Bransonic CPXH, Emerson, St. Louis, MO, USA) for 14 h and immediately after the sonication, the graphene dispersion was centrifuged for 60 min at 3000 rpm. The resulting graphene dispersion collected as the top 80% of the supernatant was characterized by UV-VIS spectroscopy (Beckman Coulter DU 720 UV/VIS Spectrophotometer, Brea, CA, USA) [33]. The concentration of LPE graphene dispersion was calculated by Lambert–Beer law [33] and it was 355 µg mL^−1^ (Figure 2).

#### 2.1.2. Liquid-Phase Exfoliated Graphene Film Fabrication

Langmuir–Blodgett technique was applied to transfer graphene thin films from the liquid–gas interface to the solid support substrate [33]. Adding a small amount of liquid-phase exfoliation (LPE) graphene dispersion into the water–air interface, the graphene nanosheets were self-organized into a close-packed film [33]. The thin and transparent film was intently scooped onto the polyethylene terephthalate (PET) substrate. After deposition, the LPE graphene film was left to dry for 20 min in ambient conditions. For the optical characterization of the liquid-phase exfoliated graphene (LPEG) films, UV-VIS spectroscopy (Beckman Coulter DU 720 UV/VIS Spectrophotometer, Brea, CA, USA) was used. The transparence of 80% was estimated for the obtained LPEG film. The transparence of the obtained LPEG film at 550 nm was estimated at 80%, which is consistent with the previously reported study [35].

### 2.2. Graphene Film Characterization

#### 2.2.1. Raman Spectroscopy of Graphene Film

Raman spectroscopy, as a noninvasive technique, has been used to provide essential information in the characterization of graphene-based materials [18,19]. Raman spectra were collected with the Micro-Raman Tri Vista 557 triple spectrometer using Nd:YAG laser (λ = 532 nm) and kept the power below 20 mW to avoid chemical damage of the film induced by the laser heating. The measurements were performed at room temperature and the acquisition time for spectra was 240 s.

#### 2.2.2. Scanning Electron Microscopy (SEM) of Graphene Film

The morphology of the LBA graphene films was characterized with scanning electron microscopy (SEM). SEM mages were obtained by Tescan MIRA3 field emission gun SEM working at 20 kV acceleration (Tescan), and SiO2/Si wafer was used as a substrate.

#### 2.2.3. Atomic Force Microscopy (AFM) of Graphene Film

Graphene film was characterized on an atomic force microscope (AFM), NTEGRA Spectra (NT-MDT). An NT MDT gold-plated tip with a nominal radius of about 30 nm was used. Scans were performed in ambient conditions, RH: 40–50%, t: 23–26 °C in semi-contact mode, with a scan frequency of 0.5 Hz and with 512 × 512 dots in the scan (10 × 10 µm surface). AFM image analysis has been performed using Gwyddionopen sourcesoftware package ver. 2.60 (Prague, Czech Republic). Thickness has been estimated at the edge of the film using profile function and statistical function in the software.

### 2.3. Cell Cultures

**Figure 3:**
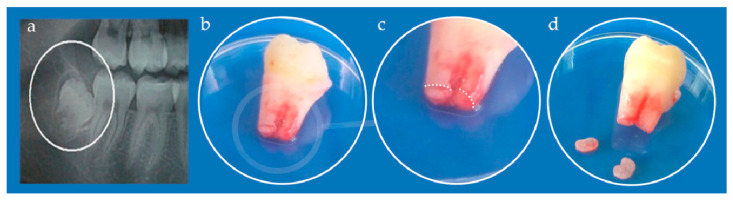
(**a**) Orthopantomogram of right mandibular impacted third molar (encircled); (**b**) Extracted tooth; (**c**) Detail from (**b**) white dotted line depicts border between apical papilla (lower parts) and tooth root (upper part); (**d**) Kidney-shaped apical papilla tissues separated from the tooth.

The study was approved by the Ethical Committee of the School of Dental Medicine, University of Belgrade (No 36/19). Immature, impacted third lower molar was extracted from a teenage patient at the Clinic for Oral Surgery (Figure 3), School of Dental Medicine, University of Belgrade, after signing the informed consents by patient’s parents. Stem cells from apical papilla were isolated as previously described [34]. Briefly, extracted tooth was rinsed with Dulbecco’s Phosphate-Buffered Saline (DPBS, Thermo Fisher Scientific, Waltham, MA, USA), and apical papilla was separated from the root apex and transferred into T-25 flasks after mincing. The tissues were grown in cell complete medium (DMEM supplemented with 10% fetal bovine serum and 1% antibiotic-antimycotic solution). Cells were cultured under standard conditions (37 °C, 95% air–5% CO_2_ atmosphere, 95% humidity) and growth medium was changed every third day. All following experiments were carried out with the cells from the fourth and fifth passage.

### 2.4. SCAP Characterization

#### 2.4.1. Flow Cytometry

Flow cytometry analyses were performed in order to assess the expression of specific mesenchymal markers of SCAP. The markers used for these analyses were: fluorescein-isothiocyanate-labeled mouse monoclonal antibodies against CD90, CD105, and CD34; phycoerythrin-labeled mouse monoclonal antibodies against CD73 and CD45 (all antibodies were purchased from Exbio, Vestec, Czech Republic). Cells were harvested with TrypLE™ Express solution, washed with DPBS supplemented with 10% FBS, and finally counted on automated cell counter Countess™ (Invitrogen, Waltham, MA, USA). One million of the cells were resuspended in 1 mLof 10% FBS solution in DPBS and incubated with adequate antibodies for 45 min in the refrigerator. After incubation, cells were fixed with 4% paraformaldehyde (PFA) for 20 min and finally rinsed 2 times with DPBS. Cells were analyzed on a tabletop flow cytometer (Partec, Munster, Germany) and results were processed by software (FloMax 2.82, Partec, Munster, Germany).

#### 2.4.2. Multilineage Differentiation Capacity

To evaluate the stemness characteristics of SCAP, their potential of differentiation into multiple lineages (osteo-, chondro- and adipo-) was tested. Cells were seeded onto 6-well plates either on PET alone or on PET coated with LPEG film, at density of 5 × 10^3^/cm^2^, and grown in the respective differentiation medium, which was changed every 2 days. After the required differentiation period of time elapsed, cells from one well were used for RNA isolation for gene expression analysis.

##### Osteo-Differentiation

After 28 days of culturing in osteo-differentiation medium (StemPro™ Osteogenesis Differentiation Kit, Thermo Fisher Scientific, Waltham, MA, USA) according to manufacturers’ recommendations, cells were subjected to histological staining method using Alizarin Red S, as previously described [34]. Briefly, after rinsing with DPBS and fixating with 4% PFA for 30 min, cells were stained with 2% Alizarin Red S (Centrohem, Belgrade, Serbia) solution, at pH 4.2. After 30 min of incubation, dye was removed, and cells were rinsed twice with distilled water. Stained cultures were observed using inverted light microscopy (Primovert, Zeiss, Oberkochen, Germany) and photographed.

##### Chondro-Differentiation

For the chondro-induction, cells were seeded in a form of micromass at total number of 1.5 × 10^6^ and grown on 6-well plates in commercially available chondrogenesis media (StemPro™ Chondrogenesis Differentiation Kit, Thermo Fisher Scientific, Waltham, MA, USA) for 21 days. Chondrogenesis was confirmed by 0.1% solution Safranin O (Centrohem, Belgrade, Serbia) positive staining. Stained cells were observed using inverted light microscopy and photographed.

##### Adipo-Differentiation

Adipogenic stimulation lasted 28 days in commercially available adipogenesis media (StemPro™ Adipogenesis Differentiation Kit, Thermo Fisher Scientific, Waltham, MA, USA) at seeding density of 1 × 10^4^ cells/cm^2^ onto 6-well plates. In order to confirm adipo-differentiation, Oil Red O (Centrohem, Belgrade, Serbia) staining was used to visualize intracellular lipid accumulation as lipid vacuoles. Stained cells were observed using inverted light microscopy and photographed.

### 2.5. LPEG Neuro-Induction

To induce neurogenic differentiation, cells (1.5 × 10^5^) were seeded onto T-25 tissue culture flasks in standard culture medium. After 24 h, neural pre-induction medium and DMEM with 100 mM beta-mercaptoethanol were added, and cells were incubated for 4 h. Then, cell differentiation was continued in a neural induction medium containing recombinant human basic fibroblast growth factor, neural growth factor, and B27 supplement (all from Thermo Fisher Scientific, Waltham, MA, USA) in DMEM either on PET alone or on PET coated with LPEG film. After 7 days of cultivation, cell morphology was observed under inverted microscope. Control cells were incubated in standard culture medium.

### 2.6. Cell Morphology Analysis Following LPEG Neuro-Induction

#### 2.6.1. Light Microscopy

Cell morphology was observed under inverted microscope (Primovert, Zeiss, Oberkochen, Germany) and photographed. Between days 3 and 7 of neurogenic culture, the cells showed a transition from fibroblast-like to neuron-like cell bodies with long processes, suggesting that the stem cells differentiated into neurons/neuron-like cells. At that point they were subjected to RNA isolation, gene expression and immunocytochemistry analysis. In addition, the growth and morphology of the cells during 5 days of LPEG neuro-induction was recorded with CytoSMART Lux 2 camera (CytoSmart Technologies BV, Eindhoven, The Netherlands).

#### 2.6.2. Confocal Microscopy

For the immunocytochemical analyses, cells were seeded onto 25 mm diameter round glass coverslips at density of 5 × 10^3^/cm^2^ and subjected to neuro-differentiation protocol as described. On the 7th day of neural induction, cells were rinsed 3 times in DPBS, fixed with 4% PFA solution for 20 min, rinsed three times with DPBS and incubated at room temperature for 45 min in blocking and permeabilization buffer (10% Bovine serum albumin and 0.1% Triton X-100 in DPBS). For immunofluorescent detection of neuronal cell marker expression, cells were incubated with the following primary antibodies: rabbit anti- β III-tubulin (B3T, 1:400, Cell Signaling, Danvers, MA, USA), rabbit anti-MAP2 (MAP 1:400, Millipore, Germany) and rabbit anti-neuronal nuclei (NeuN, 1:250, Millipore, Taufkirchen, Germany). Primary antibodies were incubated at 4 °C overnight and subsequently washed 3 times with DPBS. Cell samples were incubated with secondary antibodies—donkey anti-mouse Alexa Fluor 488 (1:200, Invitrogen, Waltham, MA, USA), donkey anti-rabbit Alexa Flour 555 (1:200, Invitrogen, Waltham, MA, USA) and donkey anti-rabbit Alexa Flour 657 (1:200, Invitrogen, Waltham, MA, USA) for 2 h in dark at room temperature. Cells were washed 3 times in DPBS and stained with 4-, 6- diamidino- 2-phenylindole (1:4000, DAPI, Molecular Probes, Eugene, OR, USA) for 10 min in dark at room temperature. After washing in DPBS cell samples were mounted with Mowiol(Sigma Aldrich, St. Louis, MO, USA) on microscope slides. Immunofluorescence microscopy images were obtained by confocal laser-scanning microscope (LSM 510, Carl Zeiss GmbH, Jena, Germany) equipped with Ar 488 and HeNe 543 and 633 laser lines. Micrographs were analyzed using Fiji-Image J softwarever 1.46 (NIH, Bethesda, MD, USA).

#### 2.6.3. AFM of Neuron-like Cells

For the purposes of atomic force microscopy, cells had to be seeded on SiO_2_ slides coated with a 2 × 2 cm graphene monolayer at a concentration of 200 cells in 10 µL of complete growth medium. The slides were placed in the wells of the 6-well plate. One hour after seeding, 740 µL of complete medium was added to the cells. After 24 h from seeding, neuro-differentiation was performed by the protocol described above.

Seven days after neuro-induction, the medium was aspirated from the well, and the plates were washed twice with DPBS, then the cells were fixed with 4% PFA solution for 20 min. Any excess fixation solution was removed by rinsing twice more with DPBS.

The morphology of the obtained cells after LPEG neuro-differentiation was characterized by microscopy on an atomic force microscope, using the same device and experimental conditions as for the graphene film characterization.

#### 2.6.4. SEM of Neuron-like Cells

After neuro-induction, cell morphology was observed by SEM using a high-resolution electron microscope, MIRA3 FEG-SEM (Tescan, Brno—Kohoutovice, Czech Republic), at a voltage acceleration of 20 kV. Cell fixation using the increscent concentrations of ethanol was done as previously described [36]. In preparation, the sample surface was coated with an ultrathin layer of gold using an SC7620 mini atomizer (Quorum Technologies, Laughton, East Sussex, UK) to prevent the accumulation of static field electricity.

### 2.7. RNA Isolation and Gene Expression

The expression of different markers was assessed by real-time PCR (qPCR) analysis. RNA was isolated using TRIzol Reagent (Thermo Fisher Scientific, Waltham, MA, USA), according to manufacturers’ recommendation. Subsequent reverse transcription from 1 μg of total RNA was performed using RevertAid First Strand cDNA Synthesis Kit (Thermo Fisher Scientific, Waltham, MA, USA)in order to obtain cDNA for qPCR analysis. The list of specific primers is given in Table 1. The results obtained from each qPCR run were threshold cycle (Ct) values. The relative expression level was assessed using the ΔΔCt method [37]. The relative mRNA expression levels for each sample were calculated as the ratio between the expression of the gene of interest and the expression of the housekeeping gene (GAPDH).

### 2.8. Statistical Analysis

GraphPad Prism ver. 9 was used for the analyses (GraphPad Software, Inc., San Diego, CA, USA). After examination of the distribution normality by Kolmogorov–Smirnov normality test, independent sample T tests were performed. The values are presented as mean ± SD. Statistical significance was set at *p* < 0.05. The experiments were performed in triplicate, repeated at least two times.

## 3. Results

### 3.1. Graphene Film Characterization

#### 3.1.1. Raman Spectroscopy of Graphene Film

**Figure 4:**
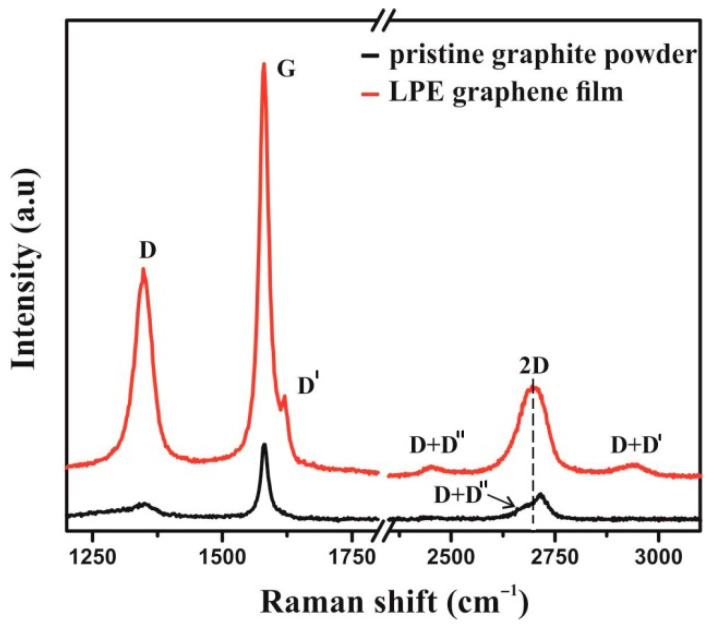
Raman spectrum of LPE graphene film (red line) and pristine graphite powder (black line).

Raman spectroscopy has been applied to verify the exfoliation of the pristine graphite powder, as bulk material, into few-layer graphene nanosheets. Figure 4 represents the Raman spectra of LPEG thin films and pristine graphite powder as a reference.

D (~1352) and G (~1582) peaks are noted in the same position at both Raman spectra. The changes of shape and Raman shift of 2D peak at Raman spectra of graphene film are evident. A well-defined and sharp shape of the 2D peak, as well as a considerable shift to lower wavenumbers (by 12 cm^−1^) compared to graphite, are characteristics of a few-layer graphene nanoflakes [24]. D′ peak (~1618 cm^−1^), visible as the shoulder of G peak in the graphene film, together with D peak, confirms the presence of defects and some amount of disorder in the graphene lattice. The combinations of the main peaks can be also observed: D + D′ (~2939 cm^−1^) and D + D″ (~2452 cm^−1^), where the D″ peak is known as a weak defect induced one phonon process.

#### 3.1.2. SEM Characterization of Graphene Film

**Figure 5:**
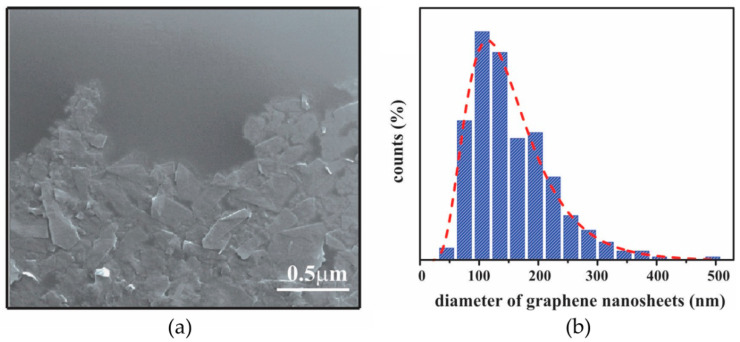
(**a**) SEM image of graphene film; (**b**) Histograms of lateral size of graphene nanosheets obtained from six 3 × 3 µm^2^ SEM images (~1800 flakes); The red dashed line represents a log-normal fit.

Information about the morphology and film structure was obtained by SEM (Figure 5). The overlapping of the graphene nanosheets and the formation of a closed packed film can be noticed in Figure 5a. Based on the measurement of lateral size, the average diameter of graphene nanosheets was estimated to be in the range of 125 ± 10 nm (Figure 5b).

#### 3.1.3. AFM Characterization of Graphene Film

**Figure 6:**
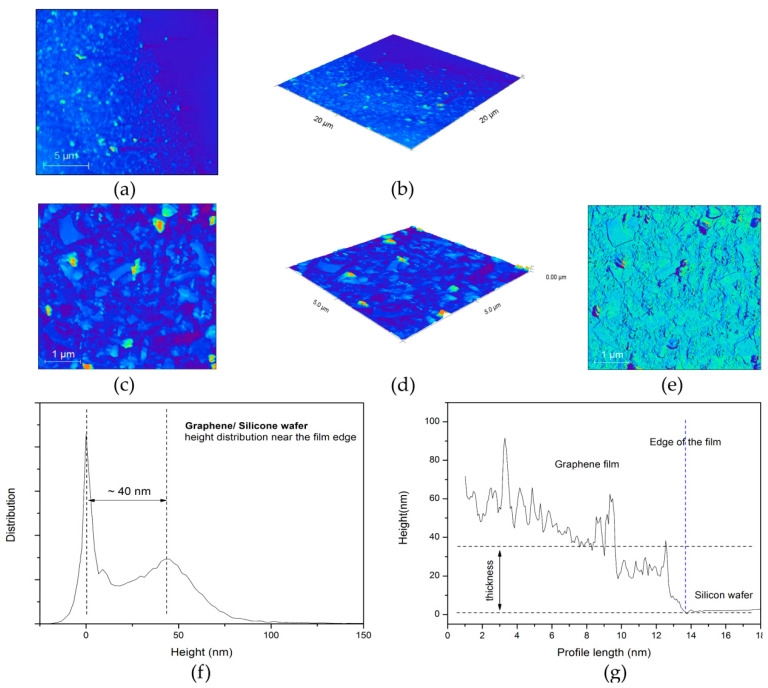
Graphene film near the edge. (**a**) 2D and (**b**) 3D image of the scan area 20 × 20 μm; (**c**) 2D, (**d**) 3D and (**e**) phase image of the scan area 5 × 5 μm; Thickness of graphene film. (**f**) Height distribution near the edge of the film measured for the area 20 × 20 μm and (**g**) average height profile across the edge of the film.

AFM scans of graphene film along with their characteristics are given in Figure 6. Both 2D (a) and 3D (b) images are shown for a scan area of 20 × 20 μm (512 × 512 lines), as well as for a scan area of 5 × 5 μm—2D image (c), 3D image (d) and phase image (e). Height distribution for the area of 20 × 20 μm and average height profile across the film are given in Figure 6f,g, respectively.

### 3.2. SCAP Characterization

#### 3.2.1. Flow Cytometry Analysis

Flowcytometry analyses were performed on P5 (fifth passage) stem cell from apical papilla. Flowcytometry revealed the expression of mesenchymal stem cell markers CD73, CD90 and CD105 (99%, 91.3% and 96%, respectively), and the absence of hematopoietic markers CD34 (0.34%) and CD45 (0.01%).

#### 3.2.2. Multilineage Differentiation Capacity

**Figure 7:**
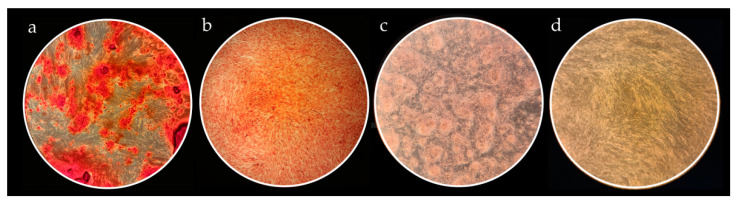
Histological evaluation ofSCAP multilineage differentiation capacity. All micrographs were taken at 40× magnification. (**a**) Alizarin Red S staining of calcium deposits showing osteogenic potential of SCAP; (**b**) Safranin O staining of proteoglycan aggregates evidencing successful SCAP chondrogenic potential; (**c**) Oil Red O positive staining of intracellular lipid droplets as a sign of SCAP adipogenicdifferention; (**d**) Representative image of unstained controls.

Alizarin Red S staining of mineralized nodules around cells confirmed osteogenic differentiation (Figure 7a); the presence of Safranin O clusters of proteoglycans characteristic for cartilage cells confirmed chondro-differentiation (Figure 7b); the presence of Oil Red O staining was indicative of intracellular lipid accumulation (Figure 7c). In the control group (non-induced cells) there were no stained cells (Figure 7d).

#### 3.2.3. Gene Expression Analysis of Multilineage Differentiation

**Figure 8:**
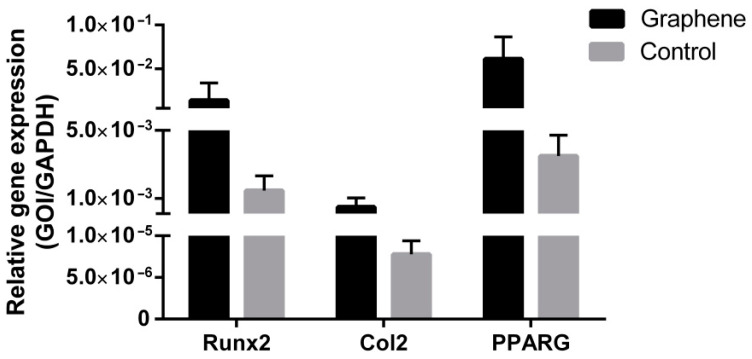
Gene expression evaluation of SCAP osteogenic (Runx2), chondrogenic (Col2) and adipogenic (PPARG) differentiation potential.

Real-time PCR analysis of gene expression confirmed successful SCAP differentiation, both when cells were grown on graphene film and when they were grown on PET only (control), thus confirming SCAP stemness. Differentiated cells grown on graphene film showed several times higher expression of Runx2—marker of bone tissue (9.59-fold increase), Col2—marker of cartilage tissue (62.90-fold increase) and PPARG—marker of adipose tissue (17.48-fold increase) compared to the control group (Figure 8), pointing to the positive effect of graphene in terms of its multilineage induction capacity.

### 3.3. LPEG Neuro-Induction of SCAP

#### 3.3.1. Light Microscopy

**Figure 9:**
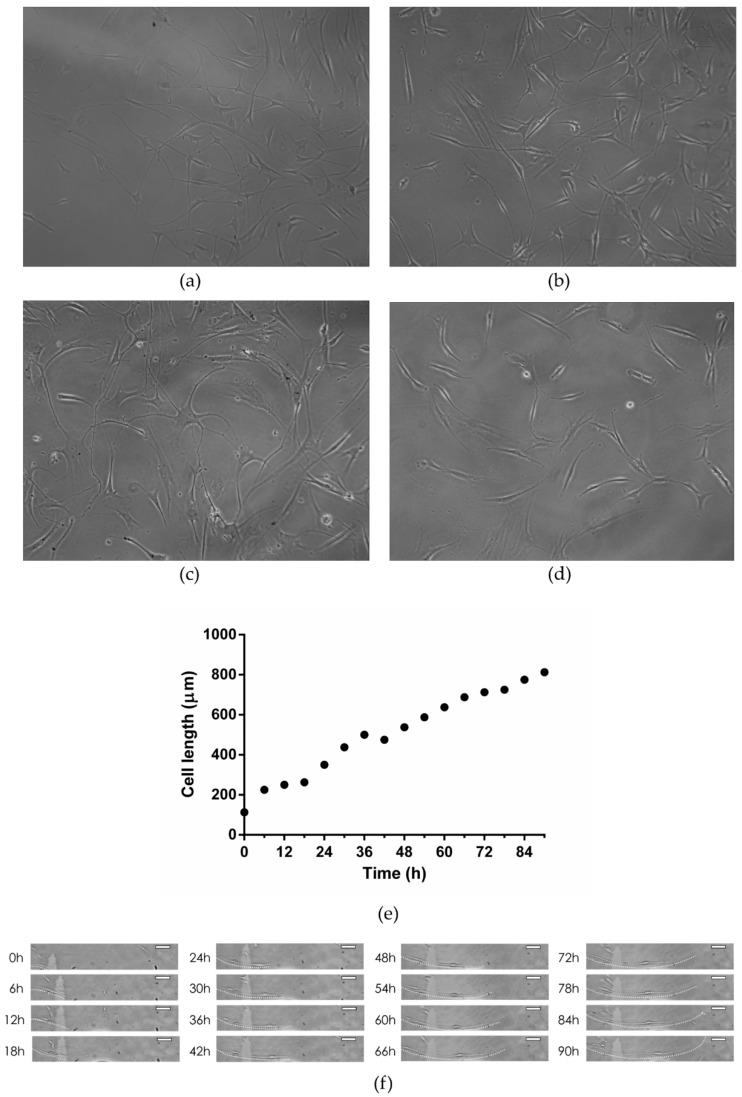
(**a**–**c**) Representative light micrographs of SCAP grown on graphene film; (**d**) Representative light micrograph of SCAP grown on PET; (**e**) Time-dependent changes in major axis length of SCAP grown on graphene film; (**f**) Time-lapse light micrographs of SCAP grown on graphene film (dotted white line represents cell extension pathway; white scale bar represents 100 µm).

After 3–5 days of neuro-induction, cells grown on LPEG film reshaped into polygonal structures with long, slender cytoplasmatic processes that were mainly in contact with adjacent cells. Representative light microscopy images of those neuron-like cells are given in Figure 9a–c. While SCAPs on LPEG film gradually changed their morphology into multipolar cells, similar to neurons, cells grown on PET showed minor changes in cell shape (Figure 9d). The growth and morphology of cells during LPEG film neuro-induction were recorded with a CytoSMART Lux 2 camera (CytoSmart Technologies BV, Eindhoven, the Netherlands). A graphical representation of the time-dependent extension of cytoplasmic processes (in µm) is shown in Figure 9e, along with 6 htime frames that were extracted from the video (Figure 9f). The real-time recording of cell morphology changes can be also viewed (Video S1).

#### 3.3.2. Confocal Microscopy

**Figure 10:**
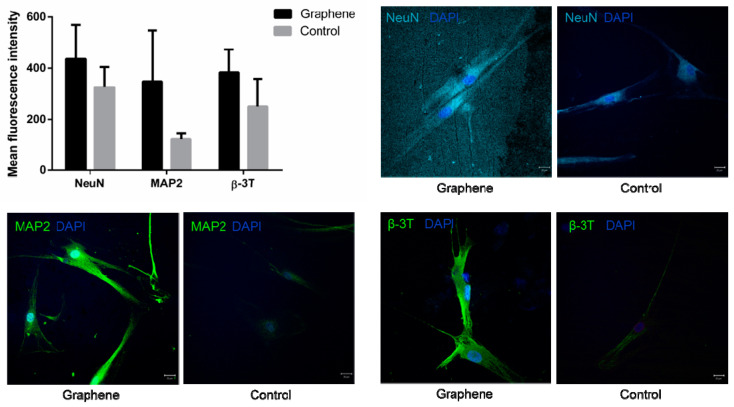
Mean fluorescence intensities and laser confocal micrographs of SCAP immunolabeled for neuronal markers NeuN, MAP2 and β3-tubulin (nuclei are labeled with DAPI).

Confocal microscopy showed the increased expression of three major neural cell markers (NeuN, MAP2 and β-3 tubulin) in SCAPs grown on graphene, compared to cells grown on PET alone (control) (Figure 10).

#### 3.3.3. AFM of Neuron-like Cells

**Figure 11:**
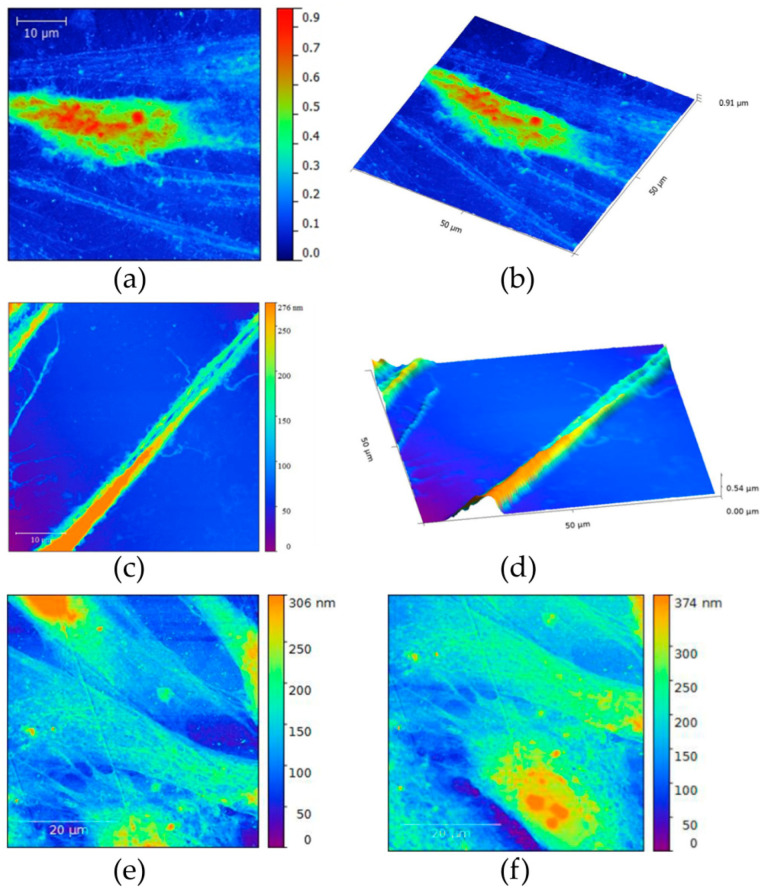
(**a**,**b**) Atomic force micrographs of SCAP grown on graphene film; (**c**,**d**) Long, slender projections of SCAP cell membrane covering graphene film; (**e**,**f**) AFMs of control SCAP grown on PET (control).

Atomic force microscopy (AFM) revealed subtle surface topography and morphological differences between stem cells grown on graphene film compared to those placed over PET. SCAP grown on graphene were polygonal in shape (Figure 11a,b) with multiple long-distance, slender cytoplasmatic projections emerging from cell body (Figure 11c,d) compared to the less complex cell morphology of SCAP grown on PET (Figure 11e,f). Note that AFM height panel also revealed numerous globular protrusions on the surface of the cell bodies, which were more present on SCAP grown on graphene.

#### 3.3.4. SEM of Neuron-like Cells

**Figure 12:**
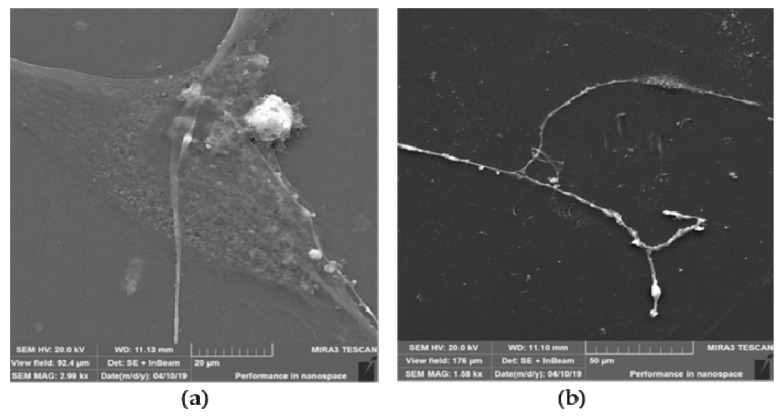
SEM of SCAP grown on graphene film. (**a**) Triangular cell body with arising long-distance membrane projections; (**b**) Slender cell projections synapsing with adjacent cell.

Scanning electron microscopy (SEM) of SCAP grown on graphene film depicted a triangular cell body with long, slender projections (Figure 12a). The endings of these projections were in close proximity or direct contact with cytoplasmatic projections of surrounding cells forming a connected cell population (Figure 12b).

#### 3.3.5. Gene Expression Analysis after LPEG Neuro-Induction

**Figure 13:**
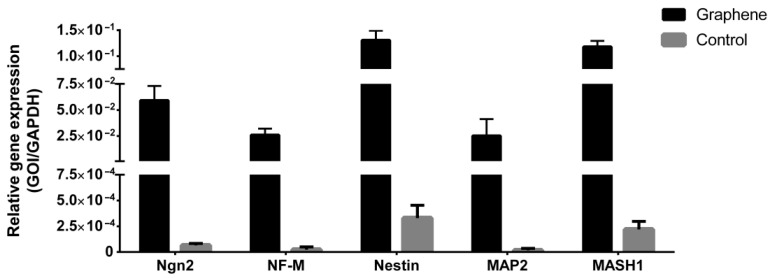
Gene expression analyses of neuronal markers of SCAP grown on graphene film and PET (control).

Gene expression analysis of key neural differentiation markers of SCAP grown on LPEG film and control material is presented in Figure 13. All examined markers showed higher expression in cells grown on graphene film compared to those on non-coated PET (control).

## 4. Discussion

Many dental tissues are precious niches of mesenchymal stem cells that are becoming increasingly appealing in regenerative medicine due to their easy accessibility and lack of health risks for the donor. They are especially attractive for the field of neuro-regeneration given that they originate from the neural crest and possess the capacity of differentiation into diverse neural cell types. Apical papilla, the soft tissue at the apex of a not fully formed tooth, contains a very high percentage of MSCs characterized by great plasticity, proliferation rate and differentiation ability. Previous studies, based on immunophenotyping, gene expression analyses, and patch clamping, have reported that SCAP grown under neural inductive conditions could give rise to a variety of neural cell phenotypes, from neuroprogenitors to mature neurons [10,34].

The number of novel materials used as cell carriers/scaffolds, tested for tissue engineering application, is constantly increasing, especially in the field of neuro-repair and regeneration. Great emphasis has been put on carbon nanostructured scaffolds that may display suitable characteristics for neural differentiation [13,34,38,39]. Graphene nanomaterials are carbon crystal allotropes with a two-dimensional structure and, according to data from the literature, have proven to be an excellent nanomaterial for neurodifferentiation due to their unique organization, chemical stability, exceptional mechanical properties, bactericidal potential, and biocompatibility [40,41]. This monoatomic layer of carbon shows the ability to absorb growth factors and exhibits electrical conductivity, which is of particular interest for the field of neuroscience. For instance, Lee et al. have convincingly demonstrated, on a neuroblastoma cell line, that graphene substrate enhanced neurite outgrowth, both in terms of length and number [42]. Rodrigues-Losada et al. also showed that different graphene materials (graphene oxide and reduced derivatives) promoted the differentiation, proliferation and maturation of dopaminergic neurons [43]. Importantly, graphene-based materials also exert stimulating effect on cell differentiation towards neurons rather than glial cells [44]. In neural regeneration, the induction of stem cell differentiation in favor of neurons against glial cells is highly desirable, making graphene-based nanomaterials a promising agent in neuroregenerative therapies. In addition to graphene oxide, the most studied graphene nanomaterial, there are other forms of graphene that are non-toxic and biocompatible, such as fully reduced or partially reduced graphene oxide, in the form of powder or film, but their positive effects in terms of neurodifferentiation have not yet been sufficiently investigated. This is the case with liquid-phase exfoliated graphene (LPEG) film that was the subject of this research. In the present study, Raman spectroscopy has been applied to verify the exfoliation of the pristine graphite powder, as bulk material, into graphene nanosheets. Indeed, the obtained closed packed film was made of few-layer graphene nanoflakes, as seen on SEM. The changes of shape and Raman shift of the 2D peak at Raman spectra of graphene film are evident. A well-defined and sharp shape of the 2D peak as well as a considerable shift to lower wave number compared to graphite are characteristics of few-layer graphene nanoflakes [45]. Edge defects, as the dominant type of defect in graphene film, are the result of the cavitation process at the liquid phase exfoliated technique [46]. Generally, the Raman spectra as well as the average diameter of the nanosheets and their height were in agreement with some previous reports [47].

In the present study, the mandatory characterization of SCAP cultures has shown a highly predominant presence of cells displaying mesenchymal stem cell markers (between 91 and 99% of cells in the culture expressed a given mesenchymal marker). Concomitantly, a negligible percentage of cells expressed hematopoietic stem cell markers (only 0.01% and 0.34% of cells expressed CD45 and CD34, respectively), as determined by flowcytometry, pointing to the fact that cell cultures contained principally MSCs. Similarly, stemness characterization by means of multiple lineages induction showed a successful osteo-, chondro- and adipo-differentiation of SCAPs. The specific osteo-, chondro- and adipo cellular phenotypes, assessed by appropriate staining procedures, were also confirmed by high mRNA levels of selected markers (Runx2, Col2 and PPARG, for osteo-, chondro- and adipo-differentiation, respectively). These findings are in general agreement with some previous reports [48,49,50]. Interestingly, the three examined processes of differentiation also appeared to be enhanced in the presence of graphene (especially chondrogenesis) but more markers specific for osteo-, chondro- and adipo- lineages should be evaluated in order to confirm that positive effect of graphene film. This is in line with some previous studies. Namely, it was found that graphene derivatives exhibit great stimulatory effects on adipogenesis and osteogenesis [19,51,52,53], pointing to the possibility of their use in composite tissue cultures when more than one cell type is needed. This is of utmost importance in regenerative medicine and dentistry when huge defects require complex reconstructions. The classical example is the surgical removal of a portion of maxilla or mandible in cases of oral cancer, resulting in massive bone, muscle and nerve defects, which necessitate multiple tissues’ replacement.

Regarding neurogenesis, the present study showed for the first time that LPEG films can have strong stimulatory effects on SCAPs’ induction towards neural lineage. Namely, cells cultured in neurodifferentiation medium on graphene film demonstrated increased levels of all neural markers (studied either by confocal microscopy or by quantitative PCR), compared to cells grown in neurodifferentiation medium only. The levels of ngn-2, an inhibitor of glial cell transcription factor, were very high, indicative of LPEG capacity to suppress gliogenesis, thus favoring neurogenesis [54]. Gene expression of Nestin, a marker of neuroepithelial and radial cells, was, as well, increased in cells grown on graphene film compared to those seeded over the non-coated substrate. This protein has a crucial role in assembling and disassembling intermediate filaments and thus maintains the structure and regulates the growth of developing neural cells [55]. Similarly, a higher expression of Mash-1, a marker of intermediate progenitors, was also noted. Mash-1, as one of the early markers that determine cellular fate, is involved in the differentiation of neuroblasts, as well as in cell protection mechanisms that prevent cell damage and apoptosis. βIII-tubulin, a neuronal microtubule protein that is particularly expressed during neurogenesis and is thought to be responsible for axon growth, was upregulated in the presence of graphene. The level of MAP2, a cytoskeletal element essential for the binding and stabilization of neuronal microtubules with major impact on neuronal development, was also higher in cells grown on graphene film [56]. Another neural marker of mature neurons that has never been found in glial cells—NeuN—was more expressed in SCAP stimulated by LPEG compared to the control condition. This marker was detected, both in the cell nucleus and perinuclear cytoplasm. Unlike the nuclear form, which binds to DNA and most probably has an important role in the regulation of neurogenesis, the role of the cytoplasmatic variant is still unclear. It is assumed that, together with Synapsin I, cytoplasmaticNeuN regulates the mobility of synaptic vesicles and release of neurotransmitters, thus playing a potential role in synaptogenesis and establishing neural circuits [57]. The last examined, final stage marker of neural development, along with MAP2 and NeuN, was Neurofilament Medium (NF-M) and, again, its expression was higher in cells grown on graphene. In agreement with our findings, which showed positive effects of graphene film on neurogenesis, a previous study that examined several types of graphene material established that the morphology of the film and the species of graphene influenced the behavior of neurons, but generally film species exhibited higher biocompatibility than powder materials [43]. Our results support the central concept of graphene substrates’ beneficial effects on the neural induction of several types of mesenchymal stem cells [58].

Future studies testing the neuroinductive capacity of graphene should use films with different physico-chemical characteristics along with other stem cells of dental origin, such as pulp or follicle cells, combined with different culture media. In addition, markers’ quantification at the protein level should rely on ELISA or Westernblot analyses as more precise than immunofluorescence quantification, thus overcoming some limitations of this study.

## 5. Conclusions

The predisposition of SCAPs to differentiate toward neural lineages, as well as the neuroinductive properties of graphene film, should warrant further studies of dental stem cells in conjunction with this nanomaterial, with the aim of finding an optimal solution for autologous neuroregenerative therapy.

## Figures and Tables

**Table 1:** Primers with corresponding sequences used in the study.

Primer Name		Sequences (5′→3′)
Runx2	Forward Reverse	ACAAACAACCACAGAACCACAAGT GTCTCGGTGGCTGGTAGTGA
Col2	Forward Reverse	TTCAGCTATGGAGATGACAATC AGAGTCCTAGAGTGACTGAG
PPARG	Forward Reverse	GCTGTGCAGGAGATCACAGA GGCTCCATAAAGTCACCAA
Ngn2	Forward Reverse	CCTGGAAACCATCTCACTTCA TACCCAAAGCCAAGAAATGC
NF-M	Forward Reverse	TGGGAAATGGCTCGTCATTT CTTCATGGAAACGGCCAA
Nestin	Forward Reverse	AACAGCGACGGAGGTCTCTA TTCTCTTGTCCCGCAGACTT
MAP2	Forward Reverse	AACCCTTTGAGAACACGACA TCTTTCCGTTCATCTGCCA
MASH1	Forward Reverse	CCAGTTGTACTTCAGCACC TGCCACTTTGAGTTTGGAC
GAPDH	Forward Reverse	TCATGACCACAGTCCATGCCATCA CCCTGTTGCTGTAGCCAAATTCGT

## Data Availability

Not applicable.

## Supplementary Materials

The following supporting information can be downloaded at: https://www.mdpi.com/article/10.3390/nano12183116/s1, Video S1: Time-dependent changes in morphology of SCAP grown on LPEG film.
